# Supplementary material for: Comparative Genomic Analyses of Virulence and Antimicrobial Resistance in Citrobacter werkmanii, an Emerging Opportunistic Pathogen
Source: Microorganisms. 2023 Aug 19;11(8):2114. doi: 10.3390/microorganisms11082114 (PMC10457828; doi:10.3390/microorganisms11082114)
Supplement: Supplementary file 1 [file microorganisms-11-02114-s001.zip › microorganisms-2531567-supplementary.pdf]

Table S1: List of *Citrobacter* reference strains used in the present study.

Citrobacter species	Strain [1]	Other strain designations	GenBank accession number
Citrobacter amalonaticus	NCTC10805^T	CCUG 4860, CECT 863, DSM 4593, ATCC 25405, CIP 82.89	UFVN01000003.1
	FDAARGOS_165		NZ_CP014070
Citrobacter braakii	ATCC 51113^T	DSM 17596, CCUG 30792, CDC 80-58, CIP 104554	NAEW01000001.1
	MiY-A		NZ_CP045771
Citrobacter cronae	Tue2-1^T		VOSQ00000000
	Colony478		NZ_CP069763
Citrobacter europaeus	97/79^T	DSM 103031, CIP 106467	GCA_900079995
	67A		PQSZ01000000
Citrobacter farmeri	GTC 1319		NZ_BBMX00000000.1
	CF_355		NZ_RHWV01000000
Citrobacter freundii	ATCC 8090^T	CIP 57.32; DSM 30039; NCTC 9750; NBRC 12681	NZ_JMTA01000001.1
	FDAARGOS_549		NZ_CP033744
	UMH13		CP024683.1
Citrobacter gillenii	CIP 106783^T	ATCC 51117; CCUG 30796; DSM 13694; CDC 4693-86	ERS574813
	UMG736		NZ_SUQN01000000
Citrobacter koseri	NCTC10786^T	ATCC 27028; CIP 82.87; DSM 4595; CCM 2537; JCM 1658	UAVY00000000
	ATCC BAA-895	4225-83	CP000822.1
	FDAARGOS_530		CP033780
Citrobacter murliniae	ATCC 51118^T	DSM 13695; CCUG 30797; CIP 104556	ERS574814
	P080C CL		GCF_004801125.1
Citrobacter pasteurii	CIP 55.13^T	BA18; DSM 28879	CDHL00000000
	UMH17		CP024676
Citrobacter portucalensis	A60^T	DSM 104542; CECT 9236	NZ_MVFY01000001.1
	FDAARGOS_617		NZ_CP044098
Citrobacter rodentium	NBRC 105723^T	DSM 16636; CIP 104675; ATCC 51116	NZ_BBNA01000001.1
	ICC168		FN543502.1
Citrobacter sedlakii	NBRC 105722^T	ATCC 51115; CCUG 30794; CIP 105037; DSM 17674	NZ_BBNB01000001.1
	3347689II		NZ_CP071070.1
Citrobacter telaviviensis	6105^T	NMI7904_11	CP045205.1
Citrobacter tructae	SNU WT2^T		NZ_CP038469
Citrobacter werkmanii	FDAARGOS_364^T	ATCC 51114; CIP 104555; CCUG 30793; DSM 17579, NBRC 105721	GCA_002386385.1
	BF-6		GCA_002025225.1
	RS189		GCA_015958985.1
	CB00044		GCA_016505055.1
Citrobacter youngae	CCUG 30791^T	CIP 105016; ATCC 29935; DSM 17578; NCTC 13709	NZ_RPOI01000016.1
	NCTC13708	CIP 55.18	UFWE00000000

[1] The superscript "T" indicates a *Citrobacter* type strain.

Table S2. Percentage of the average nucleotide identity for the *Citrobacter* strains examined in the present study.

Citrobacter species[1]	*Citrobacter werkmanii* LANIIA-032	*Citrobacter werkmanii* FDAARGOS_364^{T}	*Citrobacter freundii* ATCC 8090^{T}	*Citrobacter youngae* CCUG 30791^{T}	*Citrobacter pasteurii* CIP 55.13^{T}	*Citrobacter braakii* ATCC 51113^{T}	*Citrobacter europaeus* 97/79^{T}	*Citrobacter portucalensis* A60^{T}	*Citrobacter tructae* SNU WT2^{T}	*Citrobacter cronae* Tue2-1^{T}
Citrobacter tructae SNU WT2^{T}										66.6
Citrobacter portucalensis A60^{T}									68.3	71.1
Citrobacter europaeus 97/79^{T}								75.8	67.5	70.2
Citrobacter braakii ATCC 51113^{T}							70.3	69.7	65.6	67.0
Citrobacter pasteurii CIP 55.13^{T}						64.4	69.3	71.1	67.0	66.6
Citrobacter youngae CCUG 30791^{T}					80.4	66.7	70.9	71.9	68.2	69.1
Citrobacter freundii ATCC 8090^{T}				74.6	71.6	70.3	74.4	78.4	70.0	71.5
Citrobacter werkmanii FDAARGOS_364^{T}			74.6	70.2	67.8	69.9	73.6	73.7	69.0	79.6
Citrobacter werkmanii LANIIA-032		91.5	74.2	70.2	68.0	69.3	73.3	74.3	68.7	79.3
Citrobacter werkmanii LANIIA-031	91.4	91.1	73.9	70.5	68.3	69.0	73.3	73.8	68.6	79.2

[1] The superscript "T" indicates a *Citrobacter* type strain.
